# Knee lesions with anterior cruciate ligament (ACL) tear in Iraqi adult males: arthroscopic findings

**DOI:** 10.25122/jml-2023-0055

**Published:** 2023-09

**Authors:** Iskandar Mahdi Alardi

**Affiliations:** 1 Department of Surgery, College of Medicine, University of Al-Qadisiyah, Al Diwaniyah, Iraq

**Keywords:** ACL-associated injuries, arthroscopy, anterior cruciate ligament injury, ACLR: Anterior Cruciate Ligament Reconstruction, ACL: Anterior cruciate ligament, MCL: Medial Collateral Ligament, LCL: Lateral Collateral Ligament

## Abstract

This cross-sectional study aimed at exploring the frequency and extent of knee joint lesions associated with delayed treatment of anterior cruciate ligament (ACL) injury. It enrolled 300 patients from 2020 to 2022 who were subjected to arthroscopy for anterior cruciate ligament reconstruction. The sample was comprised of Iraqi adult male patients from different regions of Iraq, and the surgical procedure was carried out in Al-Diwaniyah Teaching Hospital. The findings were recorded by Karl Storz’s camera system. Dissection and arthroscopy were done under general anesthesia using an anterolateral portal technique. The study employed a visualization of the anterior cruciate ligament probing the meniscus and reaching the posteromedial space for the ramp lesion. The mean age of patients was 28.05±6.92 years, ranging from 19 to 35 years and the mean duration from onset of injury to the time of operation was 3.69±1.07 years, ranging from 6 months to 10 years. The arthroscopic examination revealed medial meniscus tear in 80% of the cases, lateral meniscus tear in 40% of the cases, cartilage lesion in 40% of the cases, and meniscus ramp lesions in 10% of the cases. Most cases of ACL tear are associated with a meniscus injury, cartilage defect, and collateral ligament tear. These serious lesions, such as complex meniscus tears or full articular cartilage defects, are a direct consequence of delayed treatment. Consequently, it is crucial to inform the patients about the significant issues that can arise due to treatment delays.

## INTRODUCTION

**Figure 1:**
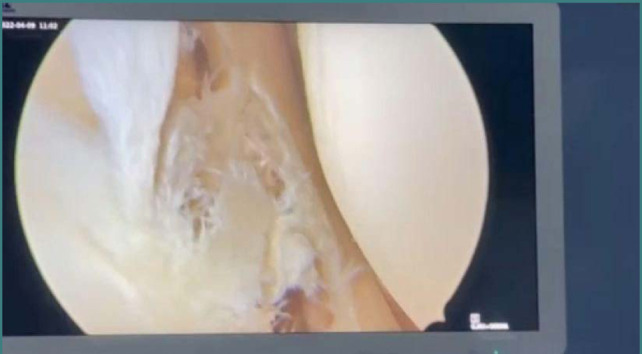
Lateral meniscus tear

**Figure 2:**
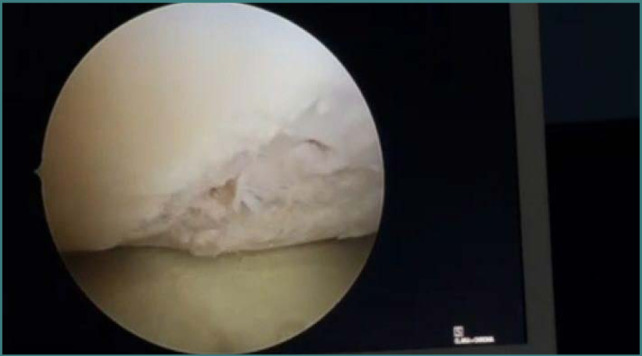
Articular loss

Anterior cruciate ligament (ACL) tear usually occurs in sports injuries or military training [1, 2]. The ACL is the stability factor for the knee joint and protects other parts of the joint such as the menisci, the medial collateral ligaments (MCL), and lateral collateral ligaments (LCL). However, most importantly, the ACL protects the articular cartilage, with a vital role in preventing the development of osteoarthritis (OA) of the joint [3-4]. In association with ACL tear, based on the available literature, the incidence of meniscus injury is the most common, followed by articular cartilage defect then by medial and lateral collateral ligament tears [5-7]. Any patient with an ACL tear should undergo surgery as soon as the range of movement is restricted, which is usually less than one month. The delay will cause more damage to the knee joint [8-13]. This research is focused on arthroscopic findings [14-16]. The presence of lesions such as meniscus tears or cartilage defects will lead to unsatisfactory results for the patients [17-19]. Even though meniscus tears usually concern the medial meniscus, the lateral meniscus can be also involved. The tear is usually variable in shape from bucket-handle to the more commonly occurring complex tear caused by multiple injuries due to the long duration since the onset of ACL tear. The cartilage defect varies from a simple fissure to a complete loss of articular surface. This is due to ACL tear, meniscus tear (Figure 1), and persistent instability that causes more articular loss usually in the medial femoral condyle (Figure 2) [5-7]. The current study aimed to explore the frequency and the extent of the knee joint lesions associated with delayed treatment of anterior cruciate ligament injuries in a sample of Iraqi adult male patients.

## MATERIAL AND METHODS

The current cross-sectional study enrolled 300 patients from 2020 to 2022 who were subjected to arthroscopy for anterior cruciate ligament reconstruction (ACLR). They were all adult males from different regions of Iraq and the surgery was done at the Al-Diwaniyah Teaching Hospital. The findings were recorded by Karl Storz’s camera system. Dissection and arthroscopy were carried out under general anesthesia using an anterolateral portal technique, to aid in the visualization of the anterior cruciate ligament, probing of the meniscus, and reaching of the posteromedial space for the ramp lesion. Data included the age of the patient, gender, weight, height, duration since onset of injury, and the findings of arthroscopy surgery. The body mass index (BMI) was calculated according to the following equation: BMI= weight in kg/(height in m)^2^. Microsoft Office Excel 2010 was used for generating descriptive statistics, such as mean, standard deviation, range, number, and percentage.

## RESULTS

The general characteristics of patients are shown in Table 1. The study included 300 male patients, with a mean age of 28.05±6.92 years with a range of 19 to 35 years. The mean duration from the onset of injury to the operation was 3.69±1.07 years, ranging from 6 months to 10 years. The mean body mass index was 21.72± 5.28kg/m^2^ and the range was from 19 to 27 kg/m^2^. The findings of the arthroscopy examination are shown in Table 2. Medial meniscus tears were present in 80% of cases, lateral meniscus tears in 40% of cases, cartilage lesions in 40% of cases, and meniscus ramp lesions in 10% of cases.

**Table 1:** General characteristics of patients

Characteristics	Results
**Age (years)**	
Range	19-35
Mean ±SD	28.05±6.92
**Gender**	
Male, n (%)	300 (100.0%)
Female, n (%)	0 (0.0%)
**BMI (kg/m^2^)**	
Range	19-27
Mean ±SD	21.72±5.28
**Duration since ACL tear (years)**	
Range	0.5-10
Mean ±SD	3.69±1.07
**Cause of injury**	
Sport, n (%)	209 (69.7%)
Military, n (%)	91 (30.3%)

n: number of cases; SD: standard deviation; BMI: body mass index; ACL: anterior cruciate ligament

**Table 2:** Types of knee joint injuries in association with ACL tear

Type of injury	Number of cases	%
Medial meniscus tear	240	80.0
Lateral meniscus tear	120	40.0
Cartilage lesion	120	40.0
Meniscus ramp lesions	30	10.0

## DISCUSSION

Reconstructions of the anterior cruciate ligament (ACL) have become increasingly common in recent years [20]. However, longitudinal studies have failed to show that ACL reconstruction can change the natural course of early-onset osteoarthritis that develops following ACL injury, raising questions about the procedure's long-term effects. The value of ACL reconstruction procedures is a topic of great interest [21]. An anterior cruciate ligament (ACL) tear can cause devastating complications such as cartilage lesions [22] and meniscus tear due to instability of the knee joint [23]. Meniscus tears will add more instability to the knee joint and increase stress on articular cartilage leading to its potential loss and the development of osteoarthritis [5-7]. In this study, most patients had a history of ACL and joint instability for more than one year. The period ranges from 6 months to 10 years after the initial injury following sports or military training which are the most frequent mechanisms of injury. Injury to the medial collateral ligament (MCL) was more common than to the lateral collateral ligament (LCL). Most ACL tears reported in the study were associated with a meniscus tear and articular cartilage defect which represents a possible secondary injury due to the long duration from the onset of injury until the time of surgery.

## CONCLUSION

The majority of ACL tears are associated with injuries to the meniscus, cartilage defects, as well as MCL and LCL tears. Most of these lesions are the result of delays in seeking treatment. A significant portion of the associated lesions are of a critical nature, such as complex meniscus tears or full articular cartilage defects. Therefore, it is essential to provide patients with thorough education regarding these severe lesions.
